# Dr. Sidney A. Pierce

**Published:** 1913-10

**Authors:** 


Dr. Sidney A. Pierce, University of Pennsylvania, 1868, died in Rochester, August 24, aged 66.

